# Hospice Administrator Perspectives on Supporting Patients after Live Discharge

**DOI:** 10.1093/geroni/igaf122.2614

**Published:** 2025-12-31

**Authors:** Kira Sheldon, Elizabeth Luth, Miriam Ryvicker, Caitlin Brennan, Yongkang Zhang

**Affiliations:** Rutgers University--New Brunswick, New Brunswick, New Jersey, United States; Rutgers University, New Brunswick, New Jersey, United States; VNS Health, New York, New York, United States; Care Dimensions, Danvers, Massachusetts, United States; Weill Cornell Medicine, New York, New York, United States

## Abstract

Live hospice discharge occurs when patients are disenrolled prior to death, impacting approximately 15% of enrollees. Addressing individuals’ needs after live discharge, particularly those at increased risk for poor outcomes, is a challenge for patients and their families and a concern for hospice agencies and the Centers for Medicare and Medicaid Services. This study examined how hospices support patients discharged alive and the potential for predictive tools identifying individuals at risk for poor post-discharge outcomes (e.g., hospitalization) to help prevent these outcomes. We analyzed interviews with 10 senior level hospice administrators in roles that impact live discharge (clinical care, data science, quality assurance, and clinical operations) at three hospices in the Northeastern and Southern United States. Participants shared that hospices support discharged patients through informal connections with healthcare providers and community organizations and formally through referral to other intra-organizational services (e.g., palliative care, GUIDE). They perceived geographic disparities in availability of post-hospice services and lack of formal infrastructure and incentives for hospices to follow-up with enrollees as contributing to poor post-discharge outcomes. They recognized the potential benefit of predictive tools to identify individuals at risk for poor post-discharge outcomes, provided concerns regarding measurement validity and end-user buy-in were adequately addressed. Careful, comprehensive discharge planning, referrals to other organization services (for larger hospices) and building connections with community services and primary care (for smaller hospices) may improve continuity of care and prevent poor outcomes following live discharge from hospice. Predictive tools may be useful if carefully specified and trusted by end-users.

